# Reliability and validity of brief psychosocial measures related to dietary behaviors

**DOI:** 10.1186/1479-5868-7-56

**Published:** 2010-07-02

**Authors:** Gregory J Norman, Jordan A Carlson, James F Sallis, Nicole Wagner, Karen J Calfas, Kevin Patrick

**Affiliations:** 1 Department of Family and Preventive Medicine, University of California, San Diego, 9500 Gilman Drive Dept 0811 La Jolla, CA 92093, USA; 2 Graduate School of Public Health, San Diego State University, San Diego, CA USA; 3 Department of Psychology, San Diego State University, San Diego, CA USA

## Abstract

**Background:**

Measures of psychosocial constructs are required to assess dietary interventions. This study evaluated brief psychosocial scales related to 4 dietary behaviors (consumption of fat, fiber/whole grains, fruits, and vegetables).

**Methods:**

Two studies were conducted. Study 1 assessed two-week reliability of the psychosocial measures with a sample of 49 college students. Study 2 assessed convergent and discriminant validity of the psychosocial measures with dietary nutrient estimates from a Food Frequency Questionnaire on 441 men and 401 women enrolled in an Internet-based weight loss intervention study.

**Results:**

Study 1 test-retest reliability ICCs were strong and ranged from .63 to .79. In study 2, dietary fat cons, fiber/whole grain cons and self-efficacy, fruit and vegetable cons and self-efficacy, and healthy eating social support, environmental factors, enjoyment, and change strategies demonstrated adequate correlations with the corresponding dietary nutrient estimates.

**Conclusions:**

Brief psychosocial measures related to dietary behaviors demonstrated adequate reliability and in most cases validity. The strongest and most consistent scales related to dietary behaviors were healthy eating change strategies and enjoyment. Consistent convergent validity was also found for the cons of change scales. These measures can be used in intervention studies to evaluate psychosocial mediators of dietary change in overweight and obese individuals.

## Background

Dietary recommendations from the U.S. Department of Health and Human Services are to consume no more than 20 to 35% of calories from fat (less than 10% of calories from saturated fat), at least 14 grams of fiber per 1000 calories consumed, and at least 2 cups of fruit and 2.5 cups of vegetables per day for a reference 2000 calorie intake [1]. However, the U.S. population eats an average of 33% of calories from fat, on the upper end of the recommended level, and an average of .8 cups of fruit and 1.8 cups of vegetables per day [2], and only 25% of adults eat 5 servings or more fruits and vegetables per day [3]. Thus, there is a need to develop more effective dietary change interventions.

Theory and empirical research should be the basis for health behavior interventions [4]. Theoretically based psychosocial constructs have been related to dietary intake in children and adolescents [5-7], overweight and obese men [8,9], and others [10-15]. Interventions targeting psychosocial constructs have had success increasing healthy dietary behaviors [16]. Though measures of psychosocial variables related to dietary behaviors have been developed for adolescents [5,17] and adults [18-21], they target a variety of dietary behaviors, have inconsistent formats, and are usually too lengthy to be used in multi-behavior studies. Brief psychosocial scales for fruit and vegetable intake have been developed but without comparable parallel scales for other dietary behaviors [22,23]. Thus, there is a need for a coordinated set of brief scales to assess a variety of theory-based psychosocial constructs related to multiple dietary outcomes.

We report two studies on the reliability and validity of brief measures of psychosocial constructs related to dietary behaviors. The psychosocial constructs were based on the Transtheoretical Model of Behavior Change [24], Social Cognitive Theory [25], and ecological models [26]. The constructs measured were self-efficacy, decisional balance (the pros and cons of change), social support, behavior change strategies (similar to the processes of change in the Transtheoretical Model), enjoyment, and neighborhood food environment. Self-efficacy and decisional balance were assessed separately for three target behaviors: total dietary fat consumption, fiber/whole grains intake, and fruit and vegetables consumption. Change strategies, social support, enjoyment and food environment were assessed for overall healthy eating.

Study 1 was conducted to determine the test-retest reliability of the psychosocial measures. Study 2 assessed evidence of convergent and discriminant validity of psychosocial measures by examining associations with dietary intake estimates. All variables were expected to be positively related to dietary intake, except for the "cons" scales, which were expected to have inverse associations. Stronger correlations between a scale and its corresponding dietary intake estimate (e.g., self-efficacy for dietary fat reduction and % energy from fat), and weaker correlations between a scale and a different dietary intake estimate (e.g., self-efficacy for dietary fat reduction and fiber grams per 1000 kcals) were considered evidence of convergent and divergent validity, respectively.

## Study 1 Methods

### Participants

Participants were college students in a large southwestern US city. The sample size was 49, ages ranged from 19 to 24 (*M *= 20.39, *SD *= 1.30), and 33 were women (67.3%). Fourteen participants identified as Asian-American/Pacific Islander (28.5%), one as Black non-Hispanic (2%), 8 as Hispanic (16.3%), and 26 as White non-Hispanic (53.1%). Body mass index (BMI) ranged from 18.4 to 31.9 (*M *= 23.29, *SD *= 2.92).

### Procedures

Participants were recruited through introductory psychology courses and received course credit. The university institutional review board approved the protocol. Participants completed identical pen and paper measures in a quiet setting at two time points across a two-week interval. After receiving directions from a research assistant that emphasized the importance of reading each item carefully, participants completed a survey booklet.

### Measures

#### Psychosocial scales

Brief psychosocial scales were developed for this study by adapting items from previously published measures [17,20,27,28] and generating new items. The goal was to develop measures for constructs that could be modified through behavior change interventions and were related to commonly targeted diet behaviors (eg, eating fruits and vegetables). However, the challenge was to measure psychosocial constructs (eg, self-efficacy) related to 4 dietary behaviors (consumption of fat, whole grains, fruits and vegetables) while limiting response burden on participants. Measures were developed for self-efficacy, decisional balance (pros of change and cons of change), and social support. We attempted to minimize response burden by collapsing fruit and vegetable consumption into one target behavior and by creating general diet-related measures of behavior change strategies, social support, food environment, and enjoyment. As a result, 9 separate scales measured dietary fat reduction self-efficacy, pros of dietary fat reduction, cons dietary fat reduction, dietary fiber self-efficacy, pros of increasing dietary fiber, cons of increasing dietary fiber, fruit & vegetable consumption self-efficacy, pros of increasing fruit & vegetable consumption, and the cons of increasing fruit & vegetable consumption. In addition, 4 scales measured social support, food environment, enjoyment, and change strategies for general healthy eating. Possible scores for each scale ranged from 1-5 where higher scores represented more of that construct. All of the psychosocial measures are located in Additional file 1.

The measure development process began by creating operational definitions of the theoretical constructs, and then 3 of the authors individually generated potential items drawing upon previously developed scales. The pool of items was then rated for face validity (ie, singularity of concept, appropriate item length, reading level). The highest rated items were retained. All scales were computed by averaging the items that comprised each scale.

Self-efficacy was assessed for increasing fruit and vegetables (6-item scale), increasing fiber and whole grains (8-item scale), and decreasing fat (5-item scale). Items were adapted from previous self-efficacy scales for eating behaviors [17,20].

The decisional balance constructs of the pros of change and cons of change for dietary intake (ie, reducing dietary fat intake, increasing fiber and whole grains, increasing fruit and vegetable servings) were adapted from previously developed measures [17]. Items on each scale related to perceptions of the positive (pros) and negative (cons) aspects of changing to healthier dietary behaviors.

The behavior change strategies for healthy eating scale was comprised of fifteen items that reflected thoughts, activities, and feelings people may use when making a behavior change. The change strategies items were based on a previously developed scale [28] with many of the items similar to the processes of change from the Transtheoretical Model [24]. Since each change strategy was only represented by a single item, a general change strategy for healthy eating score was computed rather than scales for the individual change strategies. Higher scores on this scale indicated higher frequency of using change strategies for healthy eating.

The social support for healthy eating scale assessed how often, in the past 30 days, family or friends provided support or were not supportive of eating healthy foods. Items were adapted from a previous study [29].

The food environment for healthy eating scale consisted of 4 items assessing the availability of healthy foods in the work place (e.g., There is at least one option at work where I have healthy selections to choose from) and shopping environment (e.g., There is a wide variety of fresh fruits and vegetables where I shop). Items were created by the study investigators based on published findings on food environments. Participants responded to each item on a 5-point scale from 'strongly disagree' to 'strongly agree.'

Seven items assessed enjoyment of eating what are generally considered healthy foods (e.g., I enjoy fresh fruits; I enjoy eating high fiber breakfast cereals). Participants responded to each item on a 5-point scale from 'strongly disagree' to 'strongly agree.'

### Analyses

Test-retest reliability over two weeks was evaluated with Intraclass Correlation Coefficients (ICC). ICCs assess homogeneity or stability when variables share both metric and variance [30]. Confidence intervals (95% CIs) around the ICCs indicated if the reliability estimate was statistically significant from 0 at the *p *< .05 level. Internal consistency reliability of the scales was assessed by Cronbach's alpha.

## Results

Table 1 presents means, standard deviations, and alpha coefficients for the psychosocial scales at baseline and two week follow-up. Table 1 also shows test-retest reliability estimates and 95% confidence intervals for the 13 scales. Internal consistency of the scales was generally strong at both time points, with alphas ranging between .61 and .91. Three scales had alpha values below .70: the pros and cons of dietary fat and the cons of dietary fiber/whole grains. Test-retest estimates of reliability (ICCs) for all 13 psychosocial scales were in the moderate range (.63 - .79) based on standards proposed by Shrout [31].

**Table 1:** Descriptive characteristics and reliability estimates of psychosocial scales, Study 1, N = 49.

Variable	# of items	Baseline			Two Week Follow-up			ICC	95% CI
		*M*	*SD*	alpha	*M*	*SD*	alpha		
Dietary Fat Reduction									
Pros	4	3.27	.88	.66	3.39	.85	.66	.72	.55-.83
Cons	4	2.24	.79	.64	2.37	.79	.71	.71	.54-.83
Self-efficacy	5	2.82	.82	.80	2.96	.99	.89	.70	.53-.82
Dietary Fiber and Whole Grains									
Pros	4	2.95	.89	.76	3.12	.90	.82	.73	.56-.84
Cons	4	1.84	.80	.73	1.92	.75	.74	.63	.43-.78
Self-efficacy	8	2.95	.74	.83	3.06	.86	.88	.75	.60-.85
Fruit & Vegetable									
Pros	4	3.54	.81	.73	3.57	.85	.77	.78	.64-.87
Cons	4	2.22	.83	.61	2.31	.84	.69	.74	.58-.84
Self-efficacy	6	3.12	.80	.76	3.15	.87	.81	.70	.52-.82
Healthy Eating									
Change strategies	15	3.07	.78	.91	2.99	.76	.90	.79	.66-.88
Social support	6	2.32	.85	.82	2.22	.82	.82	.68	.49-.80
Environment	4	3.60	.99	.81	3.55	.93	.83	.77	.63-.86
Enjoyment	7	3.93	.66	.72	3.90	.69	.74	.78	.65-.87

Note. *M *= mean; *SD *= standard deviation; *ICC *= intraclass correlation coefficient; *CI *= confidence interval; mean scores for each scale have a possible range of 1-5.

## Study 1 Discussion

In a university student sample, all the scales demonstrated acceptable internal consistency and test-retest reliability. The psychometric performance compared favorably to longer measures of the same constructs [28]. For example, test-retest coefficients for subscales of a longer dietary self-efficacy measure ranged from .43 to .68 [20] and from .55 to .86 for subscales of a longer social support measure [32]. The present study was limited by the well-educated sample that was mostly young and non-Hispanic white. While the study sample of college students was a sample of convenience, which by itself limits the generalizability of the findings, the study still provides important psychometric information about the psychosocial scales. However, combining these findings with Study 2, on overweight adults, will serve to test the generalizability of the scales' reliability estimates across different samples. It may also be seen as a limitation that because the scales were designed to be concise, they did not reflect the multiple factors of the constructs identified in previous studies [17,28,32,33].

## Study 2 Methods

### Participants

Women and men enrolled in separate but similar randomized controlled trials of Internet-based health promotion and weight control interventions targeting physical activity and multiple dietary outcomes. The combined sample of 842 overweight or obese adults was recruited from a large southwestern US city. The average age of participants was 42.6 (*SD *= 8.4) years and ranged from 18 to 56. Based on measured weight and height, participants' body mass index ranged from 25 to 40 (*M *= 33.31, *SD *= 4.40). The race/ethnicity distribution of the sample was 66.2% white non-Hispanic, 19.2% Hispanic, 6.2% African-American, 3.7% Asian or Pacific Islander, and 14.7% multi-ethnic or other race/ethnicities. The combined sample was 47.6% women with a majority being married (68.7%). Highest education level was distributed as 33% some college or less, 27.4% Bachelor's degree, 27.7% graduate or professional training or degree, and 11.9% some high school, high school graduate, or technical/trade school graduate.

### Procedures

The separate women's and men's randomized controlled trials were designed to evaluate 1-year behavioral weight loss intervention programs delivered mainly through the internet, with periodic email and telephone contact. The two studies used the same measurement instruments and similar data collection protocols. All measures used in the present analyses were collected at baseline, prior to participants being randomized to study conditions.

Women were recruited through their primary care providers at seven clinic sites. Participating providers sent letters to their female patients within the eligible age range informing them that they may be contacted to participate in a research study. Men were recruited from the community through newspaper and radio advertisements and posted fliers.

Eligibility criteria for both studies were for participants to have a BMI between 25 and 40, have Internet computer access, be in good general health, not be pregnant or planning to be pregnant in the next 2-years, able to read and speck English, and able to engage in moderate intensity physical activity. Potential participants were also screened for eating disorders. Participants completed survey measures on computers in a quiet setting at the research office and were compensated $15 for completing the measurement visit. All study protocols were approved by the university institutional review board, and all participants provided written informed consent.

### Measures

Participants completed the same survey measures described in Study 1. In addition, height, weight and food intake were assessed.

#### Body Mass Index (BMI)

Height and weight were measured by trained data collectors. Height was measured with a wall stadiometer, and weight was measured with a calibrated digital scale. BMI was calculated as weight in kilograms divided by height in meters squared (kg/m^2^). Two BMI categories were created based on Centers for Disease Control and Prevention (CDC) criteria wherein BMI between 25 and 34.9 equals overweight to obesity class I, and BMI between 35 and 40 equals obesity class II.

#### Food frequency questionnaire

Food frequency questionnaires (FFQ) are a widely used measure of dietary intake [34]. Participants completed the self-administered Fred Hutchinson Cancer Research Center Food Frequency Questionnaire. The measurement characteristics of this FFQ are strong and indicate similar nutrient estimates to those of 4-day food records and short-term dietary recalls [35]. This FFQ has been tested and used among multiethnic and mixed gender populations [36], and it has been shown to be useful in deriving estimates of total fruit, vegetable, fiber, and fat intake [35,37]. Participants were asked to report about foods eaten in the past month. For each food item endorsed, they indicated the usual portion size they consume. Frequency responses ranged from 'never or less than once per month' to '2 or more times per day' for foods and up to '6 or more times per day' for beverages. Portion size responses ranged from small to large and were based on a stated medium portion size and supplemented with an additional page of portion size images. Variables for the present study were calculated from the FFQ as daily percent energy from total dietary fat, daily grams of dietary fiber/1000 Kilocalories (kcals), daily servings of fruit/1000 kilocalories (kcals), and daily servings of vegetables/1000 kcals.

### Analyses

In this study, the distinction was made between dietary components (e.g., percent calories from fat) and diet behaviors (e.g., eating high-fat foods). The psychosocial scales referenced dietary behaviors and it was hypothesized that an association with the corresponding dietary intake estimate was evidence of the scales' convergent validity (e.g., dietary fat reduction self-efficacy should be associated with percent of total calories from fat). Absence of a relationship between a psychosocial scale and other dietary indicators was evidence of discriminant validity. Strength and direction of associations were determined from correlation coefficients between the psychosocial scales and the dietary components. Correlation coefficients were considered statistically significant at the p < .01 level, to partly account for multiple statistical tests. Correlations with absolute values between .11 and .12 met this criterion. To test whether validity coefficients were moderated by ethnicity, age, education, or BMI category, a series of regression models with interaction terms were specified. Potential moderators were coded as follows: ethnicity (white vs. non-white), age (median split; < 44 vs. ≥ 44), education level (< college degree vs. ≥ college degree), and BMI category (< 35 vs. ≥ 35).

## Results

Demographic characteristics for the two samples were similar. Mean age was 43.87 (SD = 7.98) for men and 41.21 (SD = 8.68) for women. BMI was slightly higher for men (Mean = 34.19; SD = 4.06) than women (Mean = 32.35; SD = 4.55). Seventy-one percent of men were white, 63% had at least a college degree, and 70% were married or living with a partner. For women, 61% were white, 46% had at least a college degree, and 67% were married or living with a partner.

The male and female samples had similar means and internal consistency alphas for each of the psychosocial scales (see Table 2). Scores were highest for dietary fat reduction pros, fiber and whole grains pros, fruit and vegetable pros, healthy eating environment, and healthy eating enjoyment (Means = 3.17 to 4.07), and lowest for dietary fat cons, fiber and whole grains cons, and fruit and vegetable cons (Means = 1.73 to 2.57). Cronbach's alphas were highest for the 3 self-efficacy scales (alphas = .83 to .89) and for healthy eating change strategies (alpha = .90) and healthy eating social support (alpha = .85).

**Table 2:** Means and standard deviations of psychosocial scales and dietary intake estimates for Study 2.

	# Items	Men (n = 441)		Women (n = 401)	
		
		Mean (SD)	alpha	Mean (SD)	alpha
Dietary Fat Reduction					
Pros	4	3.63 (.74)	.59	3.76 (.86)	.72
Cons	4	2.57 (.81)	.67	2.50 (.87)	.72
Self-efficacy	5	2.78 (.82)	.85	2.72 (.94)	.89
% energy from fat		37.7 (6.5)		35.4 (7.2)	
Dietary Fiber and Whole Grains					
Pros	4	3.11 (.87)	.73	3.57 (.95)	.80
Cons	4	1.73 (.65)	.53	1.98 (.78)	.63
Self-efficacy	8	3.27 (.77)	.86	3.17 (.86)	.89
Dietary fiber grams per 1000 kcal		8.86 (2.59)		9.28 (2.99)	
Fruit & Vegetable					
Pros	4	3.70 (.80)	.68	3.81 (.80)	.71
Cons	4	2.16 (.70)	.51	2.16 (.73)	.54
Self-efficacy	6	3.17 (.79)	.79	3.37 (.87)	.83
Fruit servings per 1000 kcal		0.59 (.46)		0.92 (.76)	
Vegetable servings per 1000 kcal		0.67 (.44)		1.24 (.91)	
Healthy Eating					
Change strategies	15	2.53 (.66)	.89	2.80 (.73)	.90
Social Support	6	2.52 (.99)	.89	2.52 (1.15)	.85
Environment	4	3.94 (.73)	.69	4.07 (.72)	.68
Enjoyment	7	3.82 (.67)	.72	3.94 (.66)	.68

Note: Scores for each scale have a possible range of 1-5.

Table 3 presents correlations among the psychosocial scales. Of the 156 correlations examined (78 for women, 78 for men), only 11 (7.1%) were above .50, and 84 (55.8%) were below .25, suggesting that each psychosocial scale was measuring a distinct construct. The self-efficacy scales were consistently moderately correlated with one another (r = .46 to .67).

**Table 3:** Correlation coefficients among psychosocial scales for study 2.

	Gender	Fat pros	Fat cons	Fat S.E.	Fiber pros	Fiber cons	Fiber S.E.	F.V. pros	F.V. cons	F.V. S.E.	H.E. strats	H.E. ss	H.E. env
Fat pros	Men												
	Women												
Fat cons	Men	.10											
	Women	.09											
Fat S.E.	Men	.11	-.27**										
	Women	.24**	-.37**										
Fiber pros	Men	.46**	-.05	.29**									
	Women	.53**	-.01	.32**									
Fiber cons	Men	.10	.24**	-.07	.10								
	Women	.02	.36**	-.20**	.02								
Fiber S.E.	Men	.16*	-.14*	.46**	.41**	-.27**							
	Women	.32**	-.22**	.64**	.55**	-.33**							
F.V. pros	Men	.51**	.07	.22**	.62**	.04	.31**						
	Women	.55**	.04	.24**	.65**	.06	.39**						
F.V. cons	Men	.08	.39**	-.15**	.03	.45**	-.23**	.09					
	Women	-.00	.49**	-.34**	-.01	.50**	-.19**	.07					
F.V. S.E.	Men	.14*	-.14*	.46**	.34**	-.18**	.64**	.30**	-.24**				
	Women	.24**	-.27**	.66**	.38**	-.22**	.67**	.37**	-.33**				
H.E. strats	Men	.33**	-.21**	.34**	.33**	-.09	.33**	.32**	-.11	.32**			
	Women	.29**	-.16*	.37**	.35**	-.24**	.48**	.29**	-.18**	.38**			
H.E. ss	Men	.33**	-.07	.20**	.22**	-.09	.24**	.37**	-.10	.26**	.53**		
	Women	.30**	-.09	.22**	.23**	-.13*	.30**	.31**	-.12	.23**	.55**		
H.E. env	Men	.10	-.05	.13*	.11	-.20**	.25**	.14*	-.18**	.20**	.19**	.14*	
	Women	.13	-.03	.12	.20**	-.24**	.27**	.14*	-.25**	.24**	.33**	.24**	
H.E. enj	Men	.14*	-.17**	.17**	.23**	-.35**	.44**	.21**	-.22**	.34**	.32**	.19**	.29**
	Women	.24**	-.20**	.27**	.41**	-.32**	.54**	.29**	-.23**	.41**	.44**	.21**	.27**

Note. * *p *< .01; ** *p *< .001; S.E. = self-efficacy; F.V. = fruit and vegetables; H.E. = health eating; strats = strategies; ss = social support; env = environment; enj = enjoyment.

Table 4 presents correlations between the psychosocial scales and dietary intake estimates. Dietary fat cons (r = .17 and .22), fiber and whole grain cons (r = -.22 and -.25), and fruit and vegetable cons (r = -.18 to -.27) were significantly related to the corresponding outcome measure for convergent validity. Dietary fat cons, fiber and whole grains cons, and fruit and vegetable cons were also related to other dietary outcomes (r = .11 to .24). However, the correlations for convergent validity were typically larger than those for discriminant validity.

**Table 4:** Correlation coefficients between psychosocial scales and dietary intake estimates for study 2.

		% energy from fat		Fiber grams per 1000 kcal		Fruit servings per 1000 kcal		Vegetable servings per 1000 kcal	
		
	Sample	r	95% CI	r	95% CI	r	95% CI	r	95% CI
Dietary Fat Reduction									
Pros	Men	-.05	-.14, .04	.05	-.04, .14	.05	-.04, .14	.04	-.05, .13
	Women	-.08	-.18, .02	.06	-.04, .16	.11	.01, .21	-.04	-.14, .06
Cons	Men	.17**	.08, .26	-.15*	-.24, -.06	-.16*	-.25, -.07	-.17**	-.26, -.08
	Women	.22**	.12, .31	-.20**	-.29, -.10	-.17*	-.26, -.07	-.17**	-.26, -.07
Self-efficacy	Men	-.07	-.16, .02	.10	.01, .19	.10	.01, .19	.15*	.06, .24
	Women	-.08	-.18, .02	.19**	.09, .28	.20**	.10, .29	.24**	.15, .33
Dietary Fiber and Whole Grains									
Pros	Men	.01	-.08, .10	.02	-.07, .11	.01	-.08, .10	.08	-.01, .17
	Women	-.12	-.22, -.02	.11	.01, .21	.14*	.04, .23	.06	-.04, .16
Cons	Men	.15*	.06, .24	-.22**	-.31, -.13	-.11	-.20, -.02	-.12*	-.21, -.03
	Women	.15*	.05, .24	-.25**	-.34, -.16	-.17*	-.26, -.07	-.16*	-.25, -.06
Self-efficacy	Men	-.01	-.10, .08	.24**	.15, .33	.11	.02, .20	.28**	.19, .36
	Women	-.11	-.21, -.01	.24**	.15, .33	.27**	.18, .36	.24**	.15, .33
Fruit & Vegetable									
Pros	Men	.06	-.03, .15	-.05	-.14, .04	-.01	-.10, .08	.08	-.01, .17
	Women	-.02	-.12, .08	.06	-.04, .16	.15*	.05, .24	.03	-.07, .13
Cons	Men	.14*	.05, .23	-.18**	-.27, -.09	-.18**	-.27, -.09	-.27**	-.35, -.18
	Women	.20**	.10, .29	-.24**	-.33, -.15	-.23**	-.32, -.14	-.20**	-.29, -.10
Self-efficacy	Men	-.10	-.19, -.01	.19**	.10, .28	.25**	.16, .34	.28**	.19, .36
	Women	-.12	-.22, -.02	.24**	.15, .33	.30**	.21, .39	.30**	.21, .39
Healthy Eating									
Change strategies	Men	-.14*	-.23, -.05	.25**	.16, .34	.21**	.12, .30	.27**	.18, .35
	Women	-.19**	-.28, -.09	.31**	.22, .40	.24**	.15, .33	.30**	.21, .39
Social support	Men	-.18**	-.27, -.09	.19**	.10, .28	.17**	.08, .26	.26**	.17, .35
	Women	-.13*	-.23, -.03	.16*	.06, .25	.19**	.09, .28	.10	.00, .20
Environment	Men	.00	-.09, .09	.13*	.04, .22	.03	-.06, .12	.19**	.10, .28
	Women	-.04	-.14, .06	.13*	.03, .23	.17*	.07, .26	.14*	.04, .23
Enjoyment	Men	-.18**	-.27, -.09	.37**	.29, .45	.22**	.13, .31	.28**	.19, .36
	Women	-.36**	-.44, -.27	.35**	.26, .43	.28**	.19, .37	.23**	.14, .32

Note. * *p *< .01; ** *p *< .001

The pros of change scales consistently were not correlated with their corresponding dietary component measures (e.g. pros of dietary fat reduction and % energy from fat, r = .05), with the exception of pros of fruit and vegetable consumption and fruit servings/1000kcals (r = .15). Dietary fat self-efficacy was not related to percent energy consumed by fat (r = -.07 and -.08), but was related to fiber and whole grain grams (r = .19) and fruit servings (r = .20) in women, and vegetable servings (r = .15 and r = .24) in both men and women. Fiber and whole grains self-efficacy (r = .24 and .24) and fruit and vegetable self-efficacy (r = .25 to .30) were related to their corresponding dietary measures. The healthy eating change strategies (|r| = .14 to .31) and enjoyment (|r| = .18 to .37) scales were the strongest correlates of dietary outcomes across the board. Healthy eating social support (|r| = .10 to .26) was also related to each of the dietary outcome measures, while healthy eating environment (r = .13 to .19) was related to fiber grams, fruit servings, and vegetable servings per 1000kcal, with the exception of fruit servings in the men's sample (r = .03).

A total of 208 tests (data not shown) were analyzed to investigate interactions between psychosocial scales and their corresponding dietary outcomes in terms of ethnicity, age, education, and BMI. Of these, only 14 were significant at the p < .05 level, suggesting that the relationship between the psychosocial scales and dietary outcome measures were essentially the same for different levels of demographic and person factors. Of the 14 interactions that were found, none were observed in both the men and women samples. Therefore, interactions were not investigated further.

## Study 2 Discussion

The results of study 2 showed that theoretically-derived brief measures of psychosocial constructs related to dietary behaviors had adequate reliability and some evidence for validity. All measures demonstrated internal consistency, but convergent validity varied substantially across psychosocial measures and little evidence was found for discriminant validity. The strongest and most consistent measures related to dietary intake were the healthy eating change strategies and healthy eating enjoyment scales. Healthy eating social support, healthy eating environment, dietary fat cons, fiber and whole grains cons, and fruit and vegetable cons also showed good validity with dietary intake.

Self-efficacy scales for fiber and whole grains and fruit and vegetables did not discriminate as well between dietary outcome measures as expected. However, this could be attributed to an overlap among dietary intake measures of fiber, fruit, and vegetables. Self-efficacy for dietary fat was not related to fat consumption, but was related to fiber grams and fruit and vegetable servings for women. These associations may be related to a phenomenon reported previously in low-income populations where individuals misjudge their fat intake and believe they are eating a low fat diet because they have increased their fruit and vegetable servings [38]. The scales measuring dietary fat pros, fiber and whole grains pros, and fruit and vegetable pros also performed poorly as they were not related to any dietary outcome measures. The overall lack of discriminant validity evidence for the psychosocial scales was likely due to the dietary intake estimates being moderately correlated with each other (absolute value r's from .25 to .50).

One plausible explanation for the more consistent relationships with the psychosocial factors for general healthy eating (change strategies, social support, and enjoyment) is that respondents may have been more able to recall beliefs and actions related to global healthy eating habits rather than the specific details about each separate dietary component. It is also possible that respondents actually make decisions and behave in terms of general healthy eating (e.g., thinking about the positive aspects of improving dietary habits) rather than focusing specifically on one dietary component (e.g., the pros of increasing consumption of whole grains). However, this non-specificity hypothesis is counter to the tenets of behavior change theories [24,25], which focus on determinants of changing discrete behaviors.

Another possible explanation is that the apparent pattern of associations was not related to general versus specific referents, but is more an indication that behavior change strategies, social support, and enjoyment may simply be more generalizable correlates of dietary intake than the other constructs. Studies are needed to identify the specificity with which people think about and implement dietary changes and evaluate measures of psychosocial variables that differ in the specificity of their dietary referents.

It was unexpected that dietary fat pros, fiber and whole grains pros, and fruit and vegetable pros were not related to any of the dietary components. Similar to present findings, previous research indicated that barriers to change were more consistently related to dietary behavior than were benefits of change [39]. A plausible explanation for stronger associations between dietary behavior and cons over pros is that the cons items tend to be more immediate and salient (e.g., cost and taste) while pros items tend to be more distant and less tangible (e.g., doing something healthy for one's body). Consistent with the Transtheoretical Model, the pros of change may influence intentions to change behaviors while cons may become more relevant once a person actually attempts to change behavior. This is consistent with the high average values for the pros items (3.11 to 3.81 on a 5-point scale), indicating that participants already tended to indorse the importance of the pros of change regardless of their dietary intake. Individual item analysis of the pros items indicated some evidence that items focusing on others (e.g., People close to me would be pleased if I ate fruits and vegetables) were less related to diet intake than self-related items (e.g., I would have more energy if I ate more fruits and vegetables) (data not shown). Developing pros items that are more immediate and focus on self-related benefits may help to improve the strength of the relationship between the pros scales and dietary intake.

The psychosocial scales reliability and validity were similar among men and women. Moderator analyses revealed that the relationship between the psychosocial scales and dietary intake estimates was the same across ethnicity, age, education, and BMI, indicating the psychosocial scales performed similarly for diverse population segments.

The present study compared psychosocial measures to dietary intake estimates such as percent calories from fat, fiber grams, and fruit and vegetable servings. This study did not address dietary behaviors such as reducing portion sizes, total calorie intake or calorie reduction and could be improved by developing a set of psychosocial measures relating to these behaviors of direct relevance to weight control. Though most psychosocial and environmental scales were supported for their validity, self-efficacy for fat intake and pros of all dietary behaviors were not supported. Further development work appears to be needed, especially for the self-efficacy scale for fat intake. A limitation was that the validation sample consisted of overweight men and women enrolled in an Internet weight loss intervention, which may have resulted in a selection bias of individuals with certain attitudes and beliefs about dietary behaviors and healthy eating. The findings may not generalize to the overall adult population.

Another study limitation was that all measures used were self-report and subject to inaccuracies and biases. However, the diet intake estimates were derived from a well established FFQ. Other strengths of the study include testing the measures in multiple samples and testing if the validity evidence was moderated by multiple individual factors of interest.

Present findings concur with the literature reporting significant associations between dietary intake and behavior change constructs of social support, self-efficacy, enjoyment, and cons of change [36,39-42]. The composite change strategies for healthy eating measure was consistently related to all dietary components, suggesting that it is a simple and potentially useful explanatory variable for healthy eating. The scale incorporated items related to self-monitoring, overcoming barriers, thinking about the benefits, social support, and goal setting, all evidence-based behavioral strategies. The healthy eating enjoyment scale also demonstrated similarly strong psychometric properties. The healthy eating enjoyment scale included items that assessed enjoyment of eating low-fat meat and dairy products providing a brief measure with considerable breadth of construct, which may be pertinent to researchers who do not want to measure pros of change for specific dietary behaviors. When assessing potential intervention mediators in the context of changing multiple behaviors, these composite measures may be an appropriate alternative to using longer measures, allowing for reduced respondent burden.

## Conclusions

The present study provides evidence for the reliability and validity of brief psychosocial scales related to 4 dietary behaviors (consumption of fat, fiber/whole grains, fruits, and vegetables). Test-retest and internal consistency reliability was assessed in college students, where as internal consistency reliability and validity was assessed in overweight and obese adults. These measures can be included in dietary change and weight loss interventions to assess to what extent interventions work through these mediating constructs to influence dietary outcomes [43]. Information from such studies is particularly valuable given the rapidly increasing rates of overweight and obesity, the health risks associated with these conditions, and the need to employ more effective interventions to treat these conditions [44].

## Competing interests

The authors declare that they have no competing interests.

## Authors' contributions

GJN, JFS, and KJC developed the survey measures. GJN, JFS, and NW were involved with the design, data collection, and statistical analyses for study 1. KP, KJC, JFS, and GJN obtained funding for the studies. GJN and JAC conducted statistical analysis for study 2 and drafted the manuscript. GJN, JAC and JFS contributed to the interpretation of the results. GJN, JAC, JFS, KJC and KP contributed to editing and revision of the manuscript. All authors read and approved the final manuscript.

## Supplementary Material

Additional file 1 **Dietary psychosocial measures used for data collection in studies 1 and 2**. The Appendix contains all of the dietary psychosocial measures used for data collection in study 1 and study 2. Click here for file

## References

[B1] USDHHS USDA. Dietary guidelines for Americans 2005 6 Washington D.C.: U.S. Government Printing Office

[B2] Centers for Disease Control and Prevention (CDC) Healthy People 2010: Heart Disease and Stroke 2005

[B3] Seymour JD Yaroch AL Serdula M Blanck HM Khan LK Impact of nutrition environmental interventions on point-of-purchase behavior in adults: a review Prev Med 2004 39 Suppl 2 S108 S136 10.1016/j.ypmed.2004.04.002 15313080

[B4] Brownson RC Baker EA Leet TL Gillespie KN Evidence-Based Public Health 2003 New York: Oxford University Press, Inc

[B5] Zabinski MF Daly T Norman GJ Rupp J Calfas KJ Sallis JF Patrick K Psychosocial correlates of fruit, vegetable, and dietary fat intake among adolescent boys and girls J Am Diet Assoc 2006 106 6 814 21 10.1016/j.jada.2006.03.014 16720122

[B6] Churchill JD Galvez R Colcombe S Swain RA Kramer AF Greenough WT Exercise, experience and the aging brain Neurobiol Aging 2002 23 5 941 55 10.1016/S0197-4580(02)00028-3 12392797

[B7] Reynolds KD Yaroch AL Franklin FA Maloy J Testing mediating variables in a school-based nutrition intervention program Health Psychol 2002 21 1 51 60 10.1037/0278-6133.21.1.51 11846345

[B8] Hagler AS Norman GJ Zabinski MF Sallis JF Calfas KJ Patrick K Psychosocial Correlates of Dietary Intake Among Overweight and Obese Men Am J Health Behav 2007 31 1 3 12 17181457 10.5555/ajhb.2007.31.1.3

[B9] Finch EA Linde JA Jeffery RW Rothman AJ King CM Levy RL The effects of outcome expectations and satisfaction on weight loss and maintenance: correlational and experimental analyses--a randomized trial Health Psychol 2005 24 6 608 16 10.1037/0278-6133.24.6.608 16287407

[B10] Kuppens R Eriksen MP Adriaanse HP Nijhuis FJ Aaron JC Determinants of fat and fiber consumption in American rural energy workers Prev Med 1996 25 2 212 7 10.1006/pmed.1996.0048 8860287

[B11] Steptoe A Perkins-Porras L McKay C Rink E Hilton S Cappuccio FP Psychological factors associated with fruit and vegetable intake and with biomarkers in adults from a low-income neighborhood Health Psychol 2003 22 2 148 55 10.1037/0278-6133.22.2.148 12683735

[B12] Oenema A Tan F Brug J Short-term efficacy of a web-based computer-tailored nutrition intervention: Main effects and mediators Annals of Behavioral Medicine 2005 29 1 54 63 10.1207/s15324796abm2901_8 15677301

[B13] de VE de NJ de Vries NK Brug J Determinants of forward stage transition from precontemplation and contemplation for fruit consumption Am J Health Promot 2005 19 4 278 85 15768922 10.4278/0890-1171-19.4.278

[B14] Fuemmeler BF Masse LC Yaroch AL Resnicow K Campbell MK Carr C Wang T Williams A Psychosocial mediation of fruit and vegetable consumption in the body and soul effectiveness trial Health Psychol 2006 25 4 474 83 10.1037/0278-6133.25.4.474 16846322

[B15] Nothwehr F Snetselaar L Yang J Wu H Stage of change for healthful eating and use of behavioral strategies J Am Diet Assoc 2006 106 7 1035 41 10.1016/j.jada.2006.04.017 16815119

[B16] Ammerman AS Lindquist CH Lohr KN Hersey J The efficacy of behavioral interventions to modify dietary fat and fruit and vegetable intake: a review of the evidence Prev Med 2002 35 1 25 41 10.1006/pmed.2002.1028 12079438

[B17] Rossi SR Greene GW Rossi JS Plummer BA Benisovich SV Keller S Velicer WF Redding CA Prochaska JO Pallonen UE Meier KS Validation of decisional balance and situational temptations measures for dietary fat reduction in a large school-based population of adolescents Eat Behav 2001 2 1 1 18 10.1016/S1471-0153(00)00019-2 15001046

[B18] Edmundson E Parcel GS Feldman HA Elder J Perry CL Johnson CC Williston BJ Stone EJ Yang M Lytle L Webber L The effects of the Child and Adolescent Trial for Cardiovascular Health upon psychosocial determinants of diet and physical activity behavior Prev Med 1996 25 4 442 54 10.1006/pmed.1996.0076 8812822

[B19] Hurley KM Caulfield LE Sacco LM Sacco LM Costigan KA Dipietro JA Measurement characteristics of diet-related psychosocial questionnaires among African-American parents and their 8- to 10-year-old daughters: Results from the Girls' health Enrichment Multi-site Studies Preventive Medicine 2004 38 S34 S42 10.1016/j.ypmed.2003.05.002 15072857

[B20] Sallis JF Pinski RB Grossman RM Patterson TL Nader PR The development of self-efficacy scales for health-related diet and exercise behaviors Health Educ Res 1988 3 3 283 92 10.1093/her/3.3.283

[B21] Steptoe A Doherty S Kerry S Rink E Hilton S Sociodemographic and psychological predictors of changes in dietary fat consumption in adults with high blood cholesterol following counseling in primary care Health Psychol 2000 19 5 411 9 10.1037/0278-6133.19.5.411 11007149

[B22] Townsend MS Kaiser LL Development of a tool to assess psychosocial indicators of fruit and vegetable intake for 2 federal programs J Nutr Educ Behav 2005 37 4 170 84 10.1016/S1499-4046(06)60243-1 16029687

[B23] Townsend MS Kaiser LL Brief psychosocial fruit and vegetable tool is sensitive for the US Department of Agriculture's Nutrition Education Programs J Am Diet Assoc 2007 107 12 2120 4 10.1016/j.jada.2007.09.015 18060898

[B24] Prochaska JO Redding CA Evers KE Glanz K, Lewis FM, Rimer BK The Transtheoretical Model and Stages of Change Health Behavior and Health Education 1997 2 San Francisco: Jossey-Bass 60 84

[B25] Bandura A Vasta R Social cognitive theory Annals of child development, Vol. 6: Six theories of child development 1989 Greenwich CT: JAI 1 60

[B26] Sallis J Owen N Fisher E Glanz K, Rimer B and Viswanath K Ecological Models of Health Behavior Health Behavior and Health Education: Theory, Research, and Practice 2008 4 United States: Jossey-Bass 465 482

[B27] Saelens BE Sallis JF Wilfley DE Patrick K Cella JA Buchta R Behavioral weight control for overweight adolescents initiated in primary care Obes Res 2002 10 1 22 32 10.1038/oby.2002.4 11786598

[B28] Saelens BE Gehrman CA Sallis JF Calfas KJ Sarkin JA Caparosa S Use of self-management strategies in a 2-year cognitive-behavioral intervention to promote physical activity Behavior Therapy 2000 31 365 379

[B29] Prochaska JJ Rodgers MW Sallis JF Association of parent and peer support with adolescent physical activity Res Q Exerc Sport 2002 73 2 206 10 12092896 10.1080/02701367.2002.10609010

[B30] McGraw KO Wong SP Forming Inferences About Some Intraclass Correlation Coefficients Psychological Methods 1996 1 1 30 46 10.1037/1082-989X.1.1.30

[B31] Shrout PE Measurement reliability and agreement in psychiatry Stat Methods Med Res 1998 7 3 301 17 10.1191/096228098672090967 9803527

[B32] Sallis JF Grossman RM Pinski RB Patterson TL Nader PR The development of scales to measure social support for diet and exercise behaviors Prev Med 1987 16 6 825 36 10.1016/0091-7435(87)90022-3 3432232

[B33] Prochaska JJ Rodgers MW Sallis JF Association of parent and peer support with adolescent physical activity Res Q Exerc Sport 2002 73 2 206 10 12092896 10.1080/02701367.2002.10609010

[B34] Johnson RK Dietary intake--how do we measure what people are really eating? Obes Res 2002 10 Suppl 1 63S 8S 10.1038/oby.2002.192 12446861

[B35] Patterson R Kristal A Tinker L Carter R Bolton M Agurs-Collins T Measurement characteristics of the Women's Health Initiative Food Frequency Questionnaire Ann Epidemiol 1999 9 3 178 87 10.1016/S1047-2797(98)00055-6 10192650

[B36] Patterson RE Kristal AR Shannon J Hunt JR White E Using a brief household food inventory as an environmental indicator of individual dietary practices Am J Public Health 1997 87 2 272 5 10.2105/AJPH.87.2.272 9103109 PMC1380806

[B37] Kristal AR Curry SJ Shattuck AL Feng ZD Li S A randomized trial of a tailored, self-help dietary intervention: The Puget Sound Eating Patterns study Preventive Medicine 2000 31 4 380 9 10.1006/pmed.2000.0711 11006063

[B38] Murphy SP Kaiser LL Townsend MS Allen LH Evaluation of validity of items for a food behavior checklist J Am Diet Assoc 2001 101 7 751 756 761 10.1016/S0002-8223(01)00189-4 11478471

[B39] Havas S Treiman K Langenberg P Ballesteros M Anliker J Damron D Feldman R Factors associated with fruit and vegetable consumption among women participating in WIC J Am Diet Assoc 1998 98 10 1141 8 10.1016/S0002-8223(98)00264-8 9787720

[B40] Ievers-Landis CE Burant C Drotar D Morgan L Trapl ES Kwoh CK Social support, knowledge, and self-efficacy as correlates of osteoporosis preventive behaviors among preadolescent females J Pediatr Psychol 2003 28 5 335 45 10.1093/jpepsy/jsg023 12808010

[B41] Van Duyn MA Kristal AR Dodd K Campbell MK Subar AF Stables G Nebeling L Glanz K Association of awareness, intrapersonal and interpersonal factors, and stage of dietary change with fruit and vegetable consumption: a national survey Am J Health Promot 2001 16 2 69 78 11727591 10.4278/0890-1171-16.2.69

[B42] Tuomilehto J Lindstrom J Eriksson JG Valle TT Hamalainen J Ilanne-Parikka P Keinanen-Liukaanniemi S Laakso M Louheranta A Rastas M Salminen V Uusitupa M Prevention of type 2 diabetes mellitus by changes in lifestyle among subjects with impaired glucose tolerance N Engl J Med 2001 344 18 1343 50 10.1056/NEJM200105033441801 11333990

[B43] Kraemer HC Wilson GT Fairburn CG Agras WS Mediators and moderators of treatment effects in randomized clinical trials Arch Gen Psychiatry 2002 59 10 877 83 10.1001/archpsyc.59.10.877 12365874

[B44] USDHHS The Surgeon General's call to action to prevent and decrease overweight and obesity 2001 U.S. Department of Health and Human Services

