# Supplementary material for: Reliability and validity of brief psychosocial measures related to dietary behaviors
Source: Int J Behav Nutr Phys Act. 2010 Jul 2;7:56. doi: 10.1186/1479-5868-7-56 (PMC2911392; doi:10.1186/1479-5868-7-56)
Supplement: Additional file 1 — Dietary psychosocial measures used for data collection in studies 1 and 2. The Appendix contains all of the dietary psychosocial measures used for data collection in study 1 and study 2. [file 1479-5868-7-56-S1.DOC]

| **Fruits & Vegetables: Pros & Cons** | | | | | | | | | | | | |
| --- | --- | --- | --- | --- | --- | --- | --- | --- | --- | --- | --- | --- |
| **The following statements are different opinions about eating fruits and vegetables. Please rate HOW IMPORTANT each statement is to your decision to eat 5 fruits and vegetables a day. Use the following scale:** | | | | | | | | | | | | |
|  | | | | | | | | | | | | |
|  | | | | | | | **Extremely Important 5** | | | | | |
| **PLEASE:** | |  | | | | Very Important 4 | | | | | |  |
| *** Fill in each circle completely.** | |  | | | Moderately Important 3 | | | | | |  |  |
| *** Erase all changes completely.** | |  | | Slightly Important 2 | | | | | |  |  |  |
|  | | | Not Important 1 | | | | | |  |  |  |  |
| 1. | I would have more energy if I ate fruits and vegetables. | | | | | | | 🞅 | 🞅 | 🞅 | 🞅 | 🞅 |
| 2. | It takes too much time to prepare fruits and vegetables. | | | | | | | 🞅 | 🞅 | 🞅 | 🞅 | 🞅 |
| 3. | I would be doing something good for my body if I ate fruits and vegetables. | | | | | | | 🞅 | 🞅 | 🞅 | 🞅 | 🞅 |
| 4. | I would rather eat sweets or high fat snacks than fruits and vegetables. | | | | | | | 🞅 | 🞅 | 🞅 | 🞅 | 🞅 |
| 5. | People close to me would be pleased if I ate fruits and vegetables. | | | | | | | 🞅 | 🞅 | 🞅 | 🞅 | 🞅 |
| 6. | Fruits and vegetables do not satisfy my hunger for very long. | | | | | | | 🞅 | 🞅 | 🞅 | 🞅 | 🞅 |
| 7. | Eating more fruits and vegetables helps me manage my weight. | | | | | | | 🞅 | 🞅 | 🞅 | 🞅 | 🞅 |
| 8. | Fresh fruits and vegetables are too expensive. | | | | | | | 🞅 | 🞅 | 🞅 | 🞅 | 🞅 |

| **Fruits & Vegetables: Self Efficacy** | | | | | | | | | | | | |
| --- | --- | --- | --- | --- | --- | --- | --- | --- | --- | --- | --- | --- |
| **There are many things that can get in the way of choosing to eat 5 fruits and vegetables each day. Rate HOW CONFIDENT you are that you can do the following using the scale below.** | | | | | | | | | | | | |
|  | | | | | | | | | | | | |
|  | | | | | | | **Extremely Confident 5** | | | | | |
| **PLEASE:** | |  | | | | Very Confident 4 | | | | | |  |
| *** Fill in each circle completely.** | |  | | | Moderately Confident 3 | | | | | |  |  |
| *** Erase all changes completely.** | |  | | Somewhat Confident 2 | | | | | |  |  |  |
|  | | | Not at All Confident 1 | | | | | |  |  |  |  |
| 1. | Eat 5 servings of fruits and vegetables everyday? | | | | | | | 🞅 | 🞅 | 🞅 | 🞅 | 🞅 |
| 2. | Drink 100% fruit juice instead of soda or fruit punch? | | | | | | | 🞅 | 🞅 | 🞅 | 🞅 | 🞅 |
| 3. | Eat fruits and vegetables for a snack instead of chips or candy? | | | | | | | 🞅 | 🞅 | 🞅 | 🞅 | 🞅 |
| 4. | Eat fruits and vegetables when eating out at a restaurant? | | | | | | | 🞅 | 🞅 | 🞅 | 🞅 | 🞅 |
| 5. | Eat fruits and vegetables when I am upset or having a bad day? | | | | | | | 🞅 | 🞅 | 🞅 | 🞅 | 🞅 |
| 6. | Eat fruits and vegetables when I am at a social event? | | | | | | | 🞅 | 🞅 | 🞅 | 🞅 | 🞅 |

| **FIBER: Pros & Cons** | | | | | | | | | | | | |
| --- | --- | --- | --- | --- | --- | --- | --- | --- | --- | --- | --- | --- |
| **The following statements are different opinions about eating high fiber foods. Please rate HOW IMPORTANT each statement is to your decision to eat foods high in fiber. Use the following scale:** | | | | | | | | | | | | |
|  | | | | | | | | | | | | |
|  | | | | | | | **Extremely Important 5** | | | | | |
| **PLEASE:** | |  | | | | Very Important 4 | | | | | |  |
| *** Fill in each circle completely.** | |  | | | Moderately Important 3 | | | | | |  |  |
| *** Erase all changes completely.** | |  | | Slightly Important 2 | | | | | |  |  |  |
|  | | | Not Important 1 | | | | | |  |  |  |  |
| 1. | Eating high fiber foods fills me up so I do not over eat. | | | | | | | 🞅 | 🞅 | 🞅 | 🞅 | 🞅 |
| 2. | Cereals and breads that are high in fiber are too expensive. | | | | | | | 🞅 | 🞅 | 🞅 | 🞅 | 🞅 |
| 3. | I am doing something good for my body when I eat high fiber foods. | | | | | | | 🞅 | 🞅 | 🞅 | 🞅 | 🞅 |
| 4. | I don't like the taste or texture of high fiber foods. | | | | | | | 🞅 | 🞅 | 🞅 | 🞅 | 🞅 |
| 5. | I have more energy when I eat high fiber foods. | | | | | | | 🞅 | 🞅 | 🞅 | 🞅 | 🞅 |
| 6. | My family does not like whole grain breads and cereals. | | | | | | | 🞅 | 🞅 | 🞅 | 🞅 | 🞅 |
| 7. | I can be a good role model for others when I eat more high fiber foods. | | | | | | | 🞅 | 🞅 | 🞅 | 🞅 | 🞅 |
| 8. | It takes too much time to find and prepare high fiber foods. | | | | | | | 🞅 | 🞅 | 🞅 | 🞅 | 🞅 |

| **FIBER: Self Efficacy** | | | | | | | | | | | | |
| --- | --- | --- | --- | --- | --- | --- | --- | --- | --- | --- | --- | --- |
| **There are many things that can get in the way of choosing to eat foods high in fiber. Rate HOW CONFIDENT you are that you can do the following using the scale below.** | | | | | | | | | | | | |
|  | | | | | | | | | | | | |
|  | | | | | | | **Extremely Confident 5** | | | | | |
| **PLEASE:** | |  | | | | Very Confident 4 | | | | | |  |
| *** Fill in each circle completely.** | |  | | | Moderately Confident 3 | | | | | |  |  |
| *** Erase all changes completely.** | |  | | Somewhat Confident 2 | | | | | |  |  |  |
|  | | | Not at All Confident 1 | | | | | |  |  |  |  |
| 1. | Choose high fiber cereals over low fiber and sugary cereals? | | | | | | | 🞅 | 🞅 | 🞅 | 🞅 | 🞅 |
| 2. | Avoid foods that are low in fiber? | | | | | | | 🞅 | 🞅 | 🞅 | 🞅 | 🞅 |
| 3. | Choose high fiber foods even when you are upset and having a bad day? | | | | | | | 🞅 | 🞅 | 🞅 | 🞅 | 🞅 |
| 4. | Choose high fiber snacks instead of doughnuts or cookies? | | | | | | | 🞅 | 🞅 | 🞅 | 🞅 | 🞅 |
| 5. | Eat 5 servings of whole grains and beans everyday? | | | | | | | 🞅 | 🞅 | 🞅 | 🞅 | 🞅 |
| 6. | Choose selections with whole grains or beans when out at a restaurant? | | | | | | | 🞅 | 🞅 | 🞅 | 🞅 | 🞅 |
| 7. | Regularly eat whole grain bread? | | | | | | | 🞅 | 🞅 | 🞅 | 🞅 | 🞅 |
| 8. | Choose foods that are high in fiber when at a social event? | | | | | | | 🞅 | 🞅 | 🞅 | 🞅 | 🞅 |

| **DIETARY FAT: Pros & Cons** | | | | | | | | | | | | |
| --- | --- | --- | --- | --- | --- | --- | --- | --- | --- | --- | --- | --- |
| **The following statements are different opinions about eating foods that are high in fat. Please rate HOW IMPORTANT each statement is to your decision to eat high fat foods. Use the following scale:** | | | | | | | | | | | | |
|  | | | | | | | | | | | | |
|  | | | | | | | **Extremely Important 5** | | | | | |
| **PLEASE:** | |  | | | | Very Important 4 | | | | | |  |
| *** Fill in each circle completely.** | |  | | | Moderately Important 3 | | | | | |  |  |
| *** Erase all changes completely.** | |  | | Slightly Important 2 | | | | | |  |  |  |
|  | | | Not Important 1 | | | | | |  |  |  |  |
| 1. | Eating my favorite high fat foods is a quick way to satisfy my hunger. | | | | | | | 🞅 | 🞅 | 🞅 | 🞅 | 🞅 |
| 2. | Eating high fat foods now can mean health problems for me in the future. | | | | | | | 🞅 | 🞅 | 🞅 | 🞅 | 🞅 |
| 3. | Foods high in fat taste better than low fat foods. | | | | | | | 🞅 | 🞅 | 🞅 | 🞅 | 🞅 |
| 4. | People close to me disapprove of me eating foods that are high in fat. | | | | | | | 🞅 | 🞅 | 🞅 | 🞅 | 🞅 |
| 5. | I feel good when I'm eating the high fat foods I enjoy. | | | | | | | 🞅 | 🞅 | 🞅 | 🞅 | 🞅 |
| 6. | Eating high fat foods makes it hard to manage my weight. | | | | | | | 🞅 | 🞅 | 🞅 | 🞅 | 🞅 |
| 7. | My family and friends like me better when I am happy and eating high fat foods rather than miserable and watching what I eat. | | | | | | | 🞅 | 🞅 | 🞅 | 🞅 | 🞅 |
| 8. | I feel sluggish and heavy when I eat high fat foods. | | | | | | | 🞅 | 🞅 | 🞅 | 🞅 | 🞅 |

| **DIETARY FAT: Self-Efficacy** | | | | | | | | | | | | |
| --- | --- | --- | --- | --- | --- | --- | --- | --- | --- | --- | --- | --- |
| **There are many things that can get in the way of choosing to eat a diet low in fat. HOW CONFIDENT are you that you can choose low fat foods in each situation?** | | | | | | | | | | | | |
|  | | | | | | | | | | | | |
|  | | | | | | | **Extremely Confident 5** | | | | | |
| **PLEASE:** | |  | | | | Very Confident 4 | | | | | |  |
| *** Fill in each circle completely.** | |  | | | Moderately Confident 3 | | | | | |  |  |
| *** Erase all changes completely.** | |  | | Somewhat Confident 2 | | | | | |  |  |  |
|  | | | Not at All Confident 1 | | | | | |  |  |  |  |
| 1. | When others around you are eating high fat foods. | | | | | | | 🞅 | 🞅 | 🞅 | 🞅 | 🞅 |
| 2. | When you are craving high fat foods. | | | | | | | 🞅 | 🞅 | 🞅 | 🞅 | 🞅 |
| 3. | When you are out at a restaurant. | | | | | | | 🞅 | 🞅 | 🞅 | 🞅 | 🞅 |
| 4. | When you are upset or having a bad day. | | | | | | | 🞅 | 🞅 | 🞅 | 🞅 | 🞅 |
| 5. | When you are at a social event. | | | | | | | 🞅 | 🞅 | 🞅 | 🞅 | 🞅 |

| **HEALTHY EATING: Change Strategies** | | | | | | | | | | | | |
| --- | --- | --- | --- | --- | --- | --- | --- | --- | --- | --- | --- | --- |
| **The following are activities, thoughts, and feelings people use to help them change their dietary habits. Think of any similar experiences you may be having or have had in the past month. Then rate HOW OFTEN you do each of the following using the scale below:** | | | | | | | | | | | | |
|  | | | | | | | | | | | | |
|  | | | | | | | | **Many Times 5** | | | | |
| **PLEASE:** | |  | | | | Often 4 | | | | | |  |
| *** Fill in each circle completely.** | |  | | | Sometimes 3 | | | | | |  |  |
| *** Erase all changes completely.** | |  | | Almost Never 2 | | | | | |  |  |  |
|  | | | Never 1 | | | | | |  |  |  |  |
| 1. | I look for information about eating healthy foods. | | | | | | 🞅 | | 🞅 | 🞅 | 🞅 | 🞅 |
| 2. | I keep track of what I eat. | | | | | | 🞅 | | 🞅 | 🞅 | 🞅 | 🞅 |
| 3. | I find ways to get around the things that get in the way of eating healthy foods. | | | | | | 🞅 | | 🞅 | 🞅 | 🞅 | 🞅 |
| 4. | I think about how my surroundings affect the foods I eat (surroundings are things like fast food restaurants, vending machines, and pre-packaged foods in the store). | | | | | | 🞅 | | 🞅 | 🞅 | 🞅 | 🞅 |
| 5. | I put reminders around my house to eat healthy foods. | | | | | | 🞅 | | 🞅 | 🞅 | 🞅 | 🞅 |
| 6. | I reward myself for eating healthy foods. | | | | | | 🞅 | | 🞅 | 🞅 | 🞅 | 🞅 |
| 7. | I do things to make eating healthy foods more enjoyable. | | | | | | 🞅 | | 🞅 | 🞅 | 🞅 | 🞅 |
| 8. | I think about the benefits I will get from eating healthy foods. | | | | | | 🞅 | | 🞅 | 🞅 | 🞅 | 🞅 |
| 9. | I try to think more about the benefits of eating healthy foods and less about the hassles of eating healthy foods. | | | | | | 🞅 | | 🞅 | 🞅 | 🞅 | 🞅 |
| 10. | I say positive things to myself about eating healthy foods. | | | | | | 🞅 | | 🞅 | 🞅 | 🞅 | 🞅 |
| 11. | When I get off track from my healthy eating goals, I tell myself I can start again and get right back on track. | | | | | | 🞅 | | 🞅 | 🞅 | 🞅 | 🞅 |
| 12. | I have a friend or family member who encourages me to eat healthy foods. | | | | | | 🞅 | | 🞅 | 🞅 | 🞅 | 🞅 |
| 13. | I try different kinds of healthy foods so that I have more choices. | | | | | | 🞅 | | 🞅 | 🞅 | 🞅 | 🞅 |
| 14. | I set goals to eat healthy foods. | | | | | | 🞅 | | 🞅 | 🞅 | 🞅 | 🞅 |
| 15. | I make back-up plans to be sure I eat healthy foods. | | | | | | 🞅 | | 🞅 | 🞅 | 🞅 | 🞅 |

| **HEALTHY EATING: Social Support** | | | | | | | | | | | | |
| --- | --- | --- | --- | --- | --- | --- | --- | --- | --- | --- | --- | --- |
| **A. How often in the last 30 days has your family or friends done the following?** | | | | | | | | | | | | |
|  | | | | | | | | | | | | |
|  | | | | | | | | **Almost Always 5** | | | | |
| **PLEASE:** | |  | | | | Often 4 | | | | | |  |
| *** Fill in each circle completely.** | |  | | | Sometimes 3 | | | | | |  |  |
| *** Erase all changes completely.** | |  | | Once in Awhile 2 | | | | | |  |  |  |
|  | | | Almost Never 1 | | | | | |  |  |  |  |
| 1. | Encourage you to eat healthy foods. | | | | | | 🞅 | | 🞅 | 🞅 | 🞅 | 🞅 |
| 2. | Discuss the benefits of eating healthy foods. | | | | | | 🞅 | | 🞅 | 🞅 | 🞅 | 🞅 |
| 3. | Remind you to choose healthy foods. | | | | | | 🞅 | | 🞅 | 🞅 | 🞅 | 🞅 |
| 4. | Share ideas on healthy eating. | | | | | | 🞅 | | 🞅 | 🞅 | 🞅 | 🞅 |
| 5. | Eat healthy meals with you. | | | | | | 🞅 | | 🞅 | 🞅 | 🞅 | 🞅 |
| 6. | Complain about eating healthy foods. | | | | | | 🞅 | | 🞅 | 🞅 | 🞅 | 🞅 |

| HEALTHY EATING: Environment | | | | | | | | | | | | |
| --- | --- | --- | --- | --- | --- | --- | --- | --- | --- | --- | --- | --- |
| **Indicate how strongly you agree with each item using the following scale:** | | | | | | | | | | | | |
|  | | | | | | | | | | | | |
|  | | | | | | | | **Strongly Agree 5** | | | | |
| **PLEASE:** | |  | | | | Somewhat Agree 4 | | | | | |  |
| *** Fill in each circle completely.** | |  | | | Neutral 3 | | | | | |  |  |
| *** Erase all changes completely.** | |  | | Somewhat Disagree 2 | | | | | |  |  |  |
|  | | | Strongly Disagree 1 | | | | | |  |  |  |  |
| 1. | There is at least one option at work where I have healthy selections to choose from. | | | | | | 🞅 | | 🞅 | 🞅 | 🞅 | 🞅 |
| 2. | There is a wide variety of fresh fruits and vegetables where I shop. | | | | | | 🞅 | | 🞅 | 🞅 | 🞅 | 🞅 |
| 3. | The fruits and vegetables where I shop are at good prices. | | | | | | 🞅 | | 🞅 | 🞅 | 🞅 | 🞅 |
| 4. | The fruits and vegetables where I shop are of good quality. | | | | | | 🞅 | | 🞅 | 🞅 | 🞅 | 🞅 |

| **HEALTHY EATING: Enjoyment** | | | | | | | | | | | | |
| --- | --- | --- | --- | --- | --- | --- | --- | --- | --- | --- | --- | --- |
| **Indicate how strongly you agree with each item using the following scale:** | | | | | | | | | | | | |
|  | | | | | | | | | | | | |
|  | | | | | | | | **Strongly Agree 5** | | | | |
| **PLEASE:** | |  | | | | Somewhat Agree 4 | | | | | |  |
| *** Fill in each circle completely.** | |  | | | Neutral 3 | | | | | |  |  |
| *** Erase all changes completely.** | |  | | Somewhat Disagree 2 | | | | | |  |  |  |
|  | | | Strongly Disagree 1 | | | | | |  |  |  |  |
| 1. | I enjoy low fat (1%) or nonfat dairy products (e.g. milk, cheese, yogurt, cottage cheese). | | | | | | 🞅 | | 🞅 | 🞅 | 🞅 | 🞅 |
| 2. | I enjoy eating fresh fruits. | | | | | | 🞅 | | 🞅 | 🞅 | 🞅 | 🞅 |
| 3. | I enjoy eating fresh raw and cooked vegetables. | | | | | | 🞅 | | 🞅 | 🞅 | 🞅 | 🞅 |
| 4. | I enjoy eating whole grain breads and crackers. | | | | | | 🞅 | | 🞅 | 🞅 | 🞅 | 🞅 |
| 5. | I enjoy eating high fiber breakfast cereals. | | | | | | 🞅 | | 🞅 | 🞅 | 🞅 | 🞅 |
| 6. | I enjoy eating lean cuts of meat. | | | | | | 🞅 | | 🞅 | 🞅 | 🞅 | 🞅 |
| 7. | I enjoy eating foods containing cooked beans (e.g. kidney, pinto). | | | | | | 🞅 | | 🞅 | 🞅 | 🞅 | 🞅 |
